# Tuberculome cérébral un challenge diagnostic: à propos d’un cas et mise au point

**DOI:** 10.11604/pamj.2019.32.176.16623

**Published:** 2019-04-10

**Authors:** Patrice Niamien Koffi, Olivier Ouambi, Nizar El Fatemi, Rachid El Maaquili

**Affiliations:** 1 Service de Neurochirurgie, CHU Ibn Sina, Rabat, Maroc

**Keywords:** Tuberculome cérébral, IRM cérébrale, spectroscopie, tuberculose du système nerveux central, Maroc, Cerebral tuberculoma, brain MRI, spectroscopy, central nervous system (CNS) tuberculosis, Morocco

## Abstract

La tuberculose du système nerveux central est le deuxième site le plus fréquent après la méningite tuberculeuse. Elle est une cause majeure de morbidité et de mortalité dans les pays en développement et représente 10 à 30% des lésions expansives intracrâniennes dans ces pays contre 0,2% dans certains pays occidentaux. Le but de cet article est de présenter un cas atypique de tuberculome cérébral dans sa présentation clinique et radiologique (l'IRM cérébrale et de la spectroscopie), de faire une mise au point à partir de cette observation sur cette pathologie infectieuse. Il s'agit d'une patiente de 44 ans, sans antécédent médico-chirurgical, qui a présenté depuis un an des céphalées, compliquées 08 mois après de crises d'épilepsies partielles à généralisation secondaire et de lourdeur de l'hémicorps gauche avec des épisodes de vomissements sans trouble visuel ni fièvre ni altération de l'état général. L'examen clinique trouvait une patiente consciente GCS= 15, pupilles égales et réactives, station debout et marche possible sans anomalie, avec hémiparésie gauche 4/5 sans autres signes neurologiques. L'IRM cérébrale objectivait en séquence T1 non injectée une lésion pariétale droite sus tentoriel plurilobées iso intense mal limitée, en séquence T2 elle est hétérogène avec un liseré hyper intense et un fond hypo intense traduisant un processus charnu avec des zones de nécroses centrales et un œdème péri-lésionnel en doigt de gans en séquence FLAIR, avec une prise de contraste annulaire intense après injection de gadolinium. L'analyse de la spectroscopie était en faveur d'une tumeur gliale. La patiente fut mise sous anticonvulsivant et a bénéficié d'un abord direct avec exérèse macroscopiquement complète. L'analyse anatomopathologique était en faveur d'un tuberculome cérébral. Elle a été mise sous traitement antituberculeux avec arrêt des crises et récupération du déficit après 04 semaines. A travers ce cas nous entrevoyons le polymorphisme clinique et radiologique qu'est le tuberculome cérébral. Il est évoqué devant un faisceau d'argument clinique, biologique et radiologique mais le diagnostic de certitude reste essentiellement anatomopathologique. La prise en charge ne saurait tarder car les complications sont néfastes et de mauvais pronostic lorsqu'il est détecté tardivement.

## Abstract (en)

Central nervous system (CNS) tuberculosis is the second most common presentation after tuberculous meningitis. It is a major cause of morbidity and mortality in the developing countries, accounting for 10-30% of intracranial expansive lesions in these countries against 0.2% in some Western countries. This study aims to report an atypical clinical and radiological manifestation (brain MRI and spectroscopy) of cerebral tuberculoma in order to provide an update on this infectious disease. The study involved a 44-year old female patient with no particular previous history, with headaches persisting for 1 year and complicated by partial secondarily generalized seizures and heaviness at the left hemicorpus with episodes of vomiting without visual disorder or fever or deterioration of general condition after 8 months. Clinical examination showed conscious patient with Glasgow Coma Scale (GCS) 15, equal and reactive pupils, walking and/or standing without fault, with 4/5 left-sided hemiparesis without other neurological signs. Brain MRI objectified right parietal subtentorial plurilobulated isointense poorly differentiated lesion on T1 weighted sequence without contrast agents injection and heterogeneous lesion with hyperintense edge and hypointense fundus, suggesting fleshy process with areas of central necrosis and perilesionnal edema extending in finger-like fashion with intense annular enhancement after gadolinium injection on T2 weighted sequence. Spectroscopy showed glial tumor. The patient received anticonvulsant therapy and macroscopic complete resection. Anatomopathologic analysis showed cerebral tuberculoma. She received antituberculosis drugs with seizure stoppage and deficit recovery after 4 weeks. This study highlights the clinical and radiological polymorphism of cerebral tuberculoma. This is suspected based on laboratory tests as well as on a body of clinical and radiological evidences but definitive diagnosis is based on anatomopathologic analysis. Early management is essential because complications can be severe and result in a poor prognosis.

## Introduction

La tuberculose reste une cause majeure de morbidité et de mortalité dans les pays en développement [1-4]. La localisation extra pulmonaire au niveau du système nerveux central est le deuxième site le plus fréquent après la méningite tuberculeuse. Elle représente 10 à 30% des lésions expansives intracrâniennes dans les pays en voie de développement contre 0,2% dans certains pays occidentaux [1, 3, 5]. L'intérêt de cet article est de présenter un cas atypique de tuberculome cérébral dans sa présentation forme clinique et radiologique (l'IRM et de la spectroscopie) et de faire une mise au point à partir de cette observation sur cette pathologie infectieuse.

## Patient et observation

Nous rapportons le cas d'une patiente admise dans notre Service de Neurochirugie d'Avicennes de Rabat au Maroc après référence de la part d'un médecin généraliste d'un centre médical provincial à notre consultation. Après notre consultation le dossier a été présenté au staff hebdomadaire de neurochirurgie pour être staffé. Le dossier médical fut retenu et la patiente a été hospitalisée. Il s'agit d'une dame de 44 ans, habitante de la campagne, mère de 5 enfants, sans antécédent médico-chirugical, ni de notion de comptage tuberculeux.

**L'histoire de sa symptomatologie** révèle dans l'anamnèse des céphalées évoluant à bas bruits, d'intensité de plus en plus croissante, de siège parieto occipital depuis un 1 an associée à deux crises convulsives de type bravais Jackson droite à généralisation secondaire 08 mois après, avec quelques épisodes de vomissements sans trouble visuel ni déficit sensitivo moteur post critique. Le tout évoluant dans un contexte de conservation de l'état général et d'apyrexie. La patiente consulte chez un médecin généraliste de l'hôpital provincial qui institue un traitement anticonvulsivant (amendant ainsi les crises convulsives) et lui demande une imagerie par résonnance magnétique cérébrale (IRM cérébrale). Au vu du résultat du bilan, il nous l'adresse pour prise en charge.

**L'examen physique** a trouvé une patiente consciente, pupilles égales et réactives avec une hémiparésie gauche discrète à 4/5. La patiente était apyrétique et en bon état général. Le reste de l'examen neurologique et somatique était sans particularité.

### Les explorations paracliniques

**Le bilan biologique**: la numération formule sanguine montrait un taux hémoglobine= 11g/dl et le taux de globule blanc à 11 000 avec taux de lymphocyte et de polynucléaire neutrophile était normal ; la numération formule sanguine avec taux d'hémoglobine= 13g/dl et plaquette= 250 000. La protéine C réactive (CRP)= 17mg/ml et la vitesse de sédimentation (VS)= 22. L'ionogramme sanguin était correct. La sérologie VIH (virus de l'immuno déficience humain) était négative et le bilan inflammatoire (vitesse de sédimentation) était normal. Le quantiferon et la recherche de bacille de Koch (BK) dans les crachats n'avaient pas initialement été réalisés mais après le résultat de l'analyse anatomopathologie. Ces derniers étaient négatifs

**Bilan radiologique**: la radiographie pulmonaire était normale.

**Figure 1:**
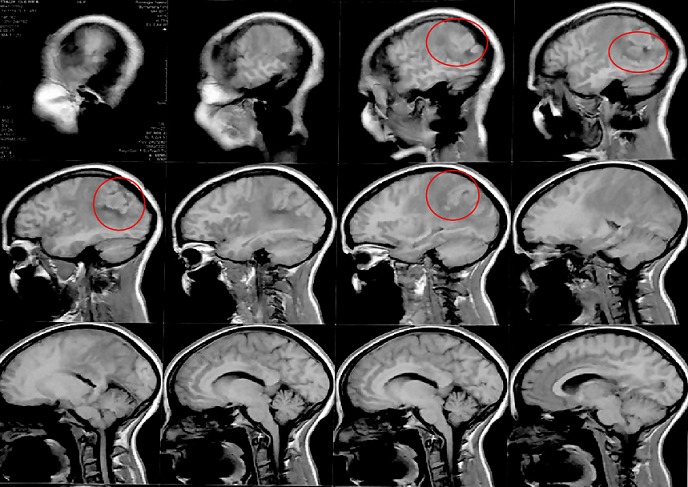
coupe sagittale en séquence t1 non injecte de l’IRM cérébrale montrant une lésion pariétale droite sus tentoriel pluri lobes iso intense mal limitée

**Figure 2:**
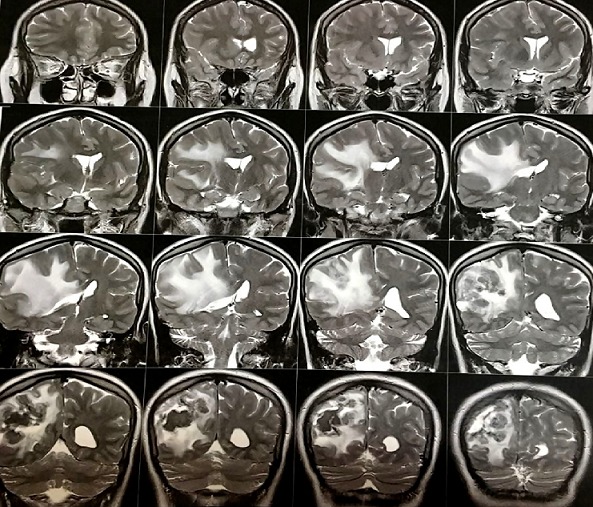
IRM coupe coronale en séquence T2 montrant un processus lésionnel pariétal droit hétérogène avec œdème péri lésionnel en doigt de gants avec un centre hypo intense témoignant d’une nécrose centrale

**Figure 3:**
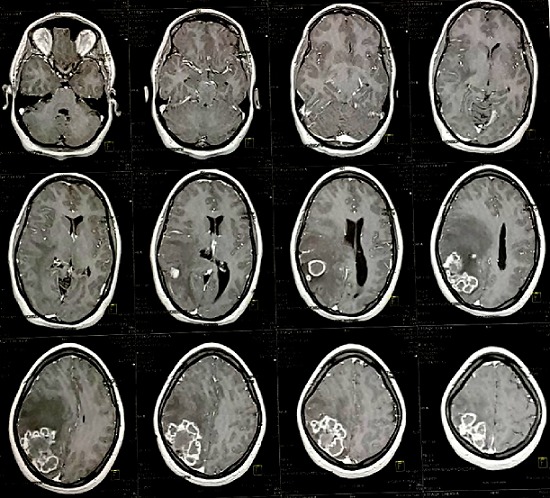
IRM coupe coronale en séquence T1 injectée montrant le processus lésionnel pariétal droit périphérique groupé en amas avec prise de contraste intense périphérique avec des zones centrales hypo intenses, zones de nécrose et un œdème réactionnel hypo intense sus et péri lésionnel responsable d’un important effet de masse sur le parenchyme controlatéral gauche et sur la ligne médiane ainsi que sur la corne occipitale du ventricule gauche

**Figure 4:**
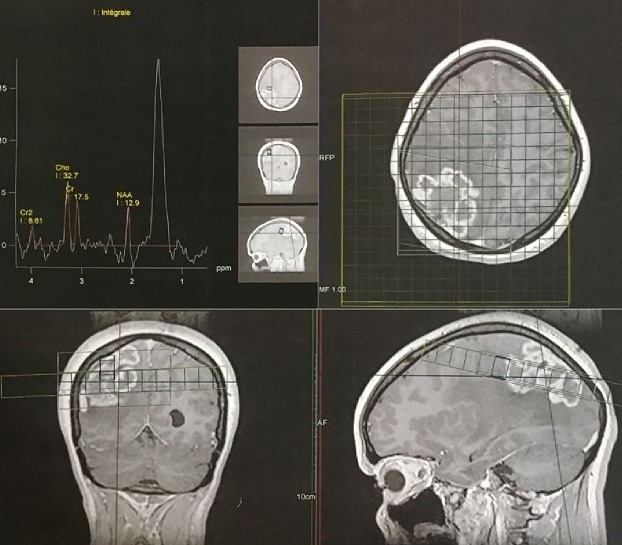
analyse spectrométrique, montre une prépondérance de la choline et N acetyl aspartate au niveau du voxel

**L'Imagerie par Résonnance Magnétique (IRM)**: l'IRM cérébrale objectivait en séquence T1 non injectée (Figure 1) une lésion pariétale droite sus tentoriel plurilobées iso intense mal limite, en séquence T2 elle est hétérogène avec un liseré hyper intense et un fond hypo intense traduisant un processus charnu avec des zones de nécroses centrales avec un œdème péri-lésionnel en doigt de gans avec une prise de contraste annulaire intense après injection de gadolinium (Figure 2 et Figure 3). L’analyse spectrométrique (Figure 4) était en faveur d'une tumeur gliale. Après analyse clinique nous avons évoqué les diagnostics suivants: 1) une tumeur gliale de haut grade; 2) une métastase cérébrale; 3) un abcès cérébral. Vu le caractère périphérique de la lésion, la patiente a bénéficié d'un abord direct avec exérèse tumorale macroscopiquement complète et le prélèvement a été envoyé en anatomopathologie. En per opératoire c'était une lésion grisâtre charnue peu hémorragique infiltrant le parenchyme par endroit dont le résultat extemporané n'a pas été formel entre une lésion gliale et un tuberculome.

**Figure 5:**
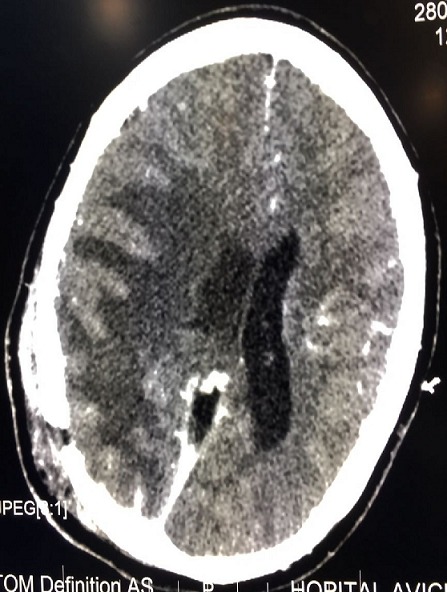
TDM de contrôle post opératoire montre la cavité poroencephalique avec volet en regard

Le résultat anatomopathologique définitif est revenu en faveur d'un tuberculome cérébral. Les suites opératoires ont été marquées par un arrêt des crises convulsives totalement après la chirurgie. La patiente a été mise sous traitement antibaccillaire avec récupération du déficit hémicorps droit au bout de quatre semaines. Un contrôle scanographique a été réalisé cinq jours après le geste chirurgical en post opératoire (Figure 5) montrant la cavité poroencephalique avec volet en regard et exérèse subtotale de la lésion. Le suivi de la patiente 06 mois après, pendant la visite en consultation externe a retrouvé une patiente consciente avec récupération totale de son déficit et disparition des crises convulsives, le reste de l'examen neurologique était normal.

## Discussion

**Physiopathologie du tuberculome cérébral**: les tuberculomes cérébraux représentent une forme particulière de la tuberculose extra-pulmonaire, plus par sa gravité potentielle que par sa fréquence [6]. La transmission du bacille tuberculeux se produit essentiellement par voie aérienne. Une fois arrivés aux alvéoles, les Bacilles de Koch sont phagocytés par les macrophages. Puis véhiculés par voie lymphatique jusqu'aux relais ganglionnaires voisins. Ils se multiplient et suscitent une réponse immunitaire qui est à l'origine de la formation des tubercules puis une caséification qui correspond à une nécrose solide dans les tissus où les bactéries se développent [7]. Lors d'analyses post-mortem, Arnold Rich et Mc Cordock ont montré qu'un foyer, appelé foyer de Rich, se développait dans le cortex cérébral ou dans les méninges, libérant, lors de sa caséification, des bacilles tuberculeux dans l'espace sous arachnoïdien. Les tuberculomes se forment quand les foyers de Rich s'élargissent sans se rompre dans les espaces sous arachnoïdiens. En effet, les tuberculomes sont souvent isolés, mais dans certains cas ils sont associés à des lésions de méningite. Les tuberculomes sont formés de cellules épithéloïdes et de cellules géantes avec des cellules lymphocytaires au centre entourant une zone de nécrose caséeuse. Le liquide de liquéfaction de la zone de nécrose est toujours clair ou de couleur « paille » par opposition au pus [1, 7]. Le tuberculome intracrânien résulte d'une diffusion hématogène à partir d'un foyer primitif généralement pulmonaire. Plusieurs tubercules se constituent puis fusionnent pour former une lésion souvent lobulée. Cette étiopathogénie explique la constitution à distance de la primo-infection tuberculeuse et l'absence souvent de Bacille de Koch (BK) dans les prélèvements [1, 7].

**Aspects cliniques et biologiques**: une étude réalisée par Faycal Moufid, à propos de 125 cas de tuberculome, colligés au service de neurochirugie du CHU IBN Sina de Rabat [1], dont la population avait un âge moyen de 26 ans [13-65] avec une prédominance féminine de 55% et la majorité des patients vivaient dans des conditions socio-économique défavorables. Dans notre cas, la patiente avait 44 ans et vivait en campagne. Les signes d'appel révélateur étaient un syndrome fébrile, une altération de l'état général dans (17%) contre 83% en bon état général comme dans notre cas parfois par un syndrome d'hypertension intracrânienne 45%, un déficit moteur dans 36%, une épilepsie dans 21% des cas, comme chez notre patiente. Une atteinte ophtalmologique a été observée chez 34 patients sous forme de baisse de l'acuité visuelle dans 15 cas [1]. Dans notre cas la patiente était consciente, en bon état général apyrétique avait présenté des crises épileptiques partielles genre bravais jacksonien avec des céphalées sans trouble visuel. La symptomatologie est non spécifique dépendant de la localisation, de la taille et du nombre des lésions. Les signes généraux (fièvre ou fébricule, asthénie, amaigrissement) dans les semaines précédant les signes neurologiques sont inconstants [1, 2]. Son mauvais pronostic impose la mise en œuvre de tous les moyens permettant d'avoir un diagnostic précoce afin de débuter rapidement un traitement spécifique. Les lésions cérébroméningées les plus fréquentes sont la leptoméningite et les tuberculomes [3]. Le tuberculome reste plus fréquent chez les patients immunodéprimés [8]. Les autres manifestations cliniques sont: 1) la méningite tuberculeuse à type de méningite fébrile avec des signes de focalisations neurologiques [4]. Le diagnostic est posé sur les résultats de la ponction lombaire après avoir éliminé une hypertension intracrânienne. Ces résultats sont une hypoglycorachie, une hyperprotéinorachie, et la lymphocytose. Par ailleurs d'autres examens peuvent aider au diagnostic, il s'agit de l'examen direct de la culture du Liquide Céphalo- Rachidien (LCR), de la réaction de polymérisation en chaine (PCR) avec recherche d'antigène. 2) L'hydrocéphalie est une autre ces manifestations fréquente et due à une obstruction d'origine inflammatoire des citernes de la base, parfois par une compression des voies d'écoulement du LCR par des tuberculomes [9]. Les autres complications peuvent être des lésions de miliaires multiples au niveau cérébral, des abcès solitaires ou nombreux à type de miliaires tuberculeuses, une pachyméningite, un infarcissement ischémique et parfois une thrombophlébite cérébrale [9]. Boukobza *et al.* ont retrouvé sur une série de 12 cas, la présence de tuberculose extra cérébrale concomitante dans 7 cas sur 12 soit (58%) et le Bacille de Koch (BK) a été retrouvé dans les crachats. Ces patients étaient de statut sérologique négatif au virus d'immunodéficience humain (VIH) [8]. Dans notre cas la recherche du VIH et du BK étaient négatives. La vitesse de sédimentation (VS) était élevée dans 80% des cas dans l'étude de Faycal Moufid et collaborateurs [1], dans notre cas la VS était normale. Le grand polymorphisme clinique et le manque de spécificité des signes radiologiques rendent le diagnostic difficile et sont fréquemment responsables d'un retard de traitement. Radiologiquement on a évoqué le diagnostic de lésion tumoral vue son caractère à l'IRM confirmer par la spectroscopie qui toute fois est non spécifique.

**Aspect IRM et spectrocopie**: l'aspect des tuberculomes cerebraux à l'IRM dépend du stade évolutif et de la présence ou non de nécrose caséeuse [10]. Les lésions revêtent différents aspects radiologiques. L'aspect typique évocateur d'un tuberculome n'est retrouvé que dans 34% des cas. Alors que dans la majorité des cas le tuberculome prend un aspect non spécifique pouvant évoquer une pathologie tumorale ou inflammatoire [10]. Une tuberculose pseudo-tumorale aurait pu être évoquée vu le contexte et l'aspect radiologique selon Boukobza *et al*. En effet selon leur étude portant sur la tuberculose du système nerveux central, aspects IRM et évolutions sur 12 cas [8] a révélé la présence de tuberculome cérébral dans 67% soit 8 patients sur 12. Concernant la localisation intra cérébrale du tuberculome (TIC), il peut siéger n'importe où dans le cerveau. Classiquement, la localisation avait plutôt tendance à être sous-tentorielle chez l'enfant, sus tentorielle chez l'adulte. Dans 15 à 20% des cas, il y avait plusieurs tuberculomes [1,11]. Pour Faycal Moufid *et al.*, la lésion était unique dans 90% des cas de siège sus-tentoriel (60%) et (40%) en sous tentoriel [1].Chez notre patiente il s'agit d'un tuberculome de siège cérébral pariétal postérieur mature groupés en foyer avec des lésions coalescentes donnant un aspect de lésions multilobées en iso signal T1 avec un centre en hyposignal en séquence T2. On note un rehaussement après injection de gadolinium. Selon Faycal, le tuberculome cérébral a l'IRM peut se présenter soit avec un signal iso-intense à hypo-intense par rapport au parenchyme cérébral en séquence pondérée T1 et hypo-intense en séquence pondérée T2 [1, 2]. Le tuberculome est entouré d'une zone irrégulière d'hypersignal en séquence T2 correspondant à un œdème. Après injection de gadolinium, on observe une prise de contraste intense périphérique et circulaire. Le tuberculome intracrânien est le plus souvent unique et les formes multiples restent rares [1]. L'aspect des tuberculomes cérébraux à l'IRM dépend du stade évolutif et de la présence ou non de nécrose caséeuse [12]. Les lésions revêtent différents aspects radiologiques. L'aspect typique évocateur d'un tuberculome n'est retrouvé que dans 34% des cas. Alors que dans la majorité des cas le tuberculome prend un aspect non spécifique pouvant évoquer une pathologie tumorale ou inflammatoire [10]. Cet aspect radiologique n'est ni constant ni spécifique pouvant évoquer de nombreuses autres pathologies inflammatoires (cysticercoses et abcès à pyogènes) ou néoplasiques (métastases, gliomes ou lymphomes) [12]. Au stade précoce, le tuberculome présente un aspect d' hypersignal discret en séquence T1 et hypo intense en séquence T2 avec rehaussement nodulaire [10, 12]. Les tuberculomes non caséifiés paraissent en hyposignal en séquence T1 et hypersignal en séquence T2 par rapport au parenchyme cérébral et se rehaussent de façon intense et homogène apres injection de produit de contraste en séquence T1. Les tuberculomes caséifiés à centre solide paraissent en hypo ou iso signal en séquence aussi bien en T1 qu'en T2 et s'associent souvent à un œdème peri-lésionnel. Les tuberculomes caséifiés à centre nécrotique paraissent en hyposignal en séquence T1 et en hyper signal en séquence T2 et se rehaussent en périphérie après injection de produit de contraste [12, 13]. L'étude spectroscopique de la lésion chez notre patiente avait montré un pic de choline avec rapport choline/NAA >2, ainsi qu'un pic de lactate. La sémiologie de l'IRM et de la spectroscopie a plaidé en faveur d'une origine tumorale en premier c'est-à-dire, un gliome de haut grade et secondairement une lésion métastatique.

**Traitement**: le traitement chirurgical dépend à la fois de la forme clinique et de la localisation cérébrale. Les indications chirurgicales sont nombreuses allant d'un abord direct avec décompression cérébrale pour une lésion corticale et comprimante hémisphérique ou de la fosse cérébrale postérieure. Une biopsie en condition stéréotaxique sera réalisée pour des formes profondes peu accessibles chirurgicalement (de la base du crane ou du tronc cérébrale) et les formes multifocales ou abcédée avec aspiration sous cadre stéréotaxique après repérage scanographique [1]. Une dérivation ventriculo péritonéale est parfois indiquée en cas de dilatation tétra ou tri ventriculaire en cas d’hypertension intracranienne et des signes de résorption transependymaire avec ou sans obstruction des voies d'écoulement du liquide cerebro spinal (LCS) et si l'aspect biochimique du LCS le permet. En plus du traitement chirurgical pour les formes kystiques, compressives et responsables de déficit ou avec hydrocéphalie le traitement médical est toujours nécessaire. La tuberculose est une maladie à déclaration obligatoire et le traitement médical bien codifié. Les méningites tuberculeuses de même que les tuberculomes cérébraux seront traitées selon (2SRHZ/7RH), c'est-à-dire: une phase initiale de 2 mois associant: streptomycine, rifampicine, isoniazide, pyrazinamide. Une phase de continuation de 7 mois associant: rifampicine et isoniazide. La durée totale du traitement est en général de 9 à 12 mois, avec toujours deux phases: une phase d'attaque associant les 4 anti-bacillaires cités précédemment et d'une durée de 2 mois, suivie d'une phase dite d'entretien allant de 7 à 10 mois et pendant laquelle seuls l'isoniazide et la rifampicine sont maintenus [14]. Diagnostiqué et pris en charge précocement comme dans notre cas le pronostic est bon mais peut être défavorable en cas d'immunodépression au VIH ou chez un patient diabétique mal équilibré ou ayant insuffisance rénale ou associée à un milieu de vie défavorable avec précarité du mode de vie.

## Conclusion

A travers ce cas nous entrevoyons le polymorphisme clinique et radiologique qu'est le tuberculome cérébral. Il est évoqué devant un faisceau d'argument clinique, radiologique et biologique mais le diagnostic de certitude reste essentiellement anatomopathologique [2, 15]. La prise en charge ne serait tarder car les complications sont néfastes [1, 3, 11, 16, 17] et de mauvais pronostic vu tardivement. Pour plusieurs auteurs une intradermo-réaction importante à la tuberculine (supérieure à 15mm) traduit une infection tuberculeuse active même en zone endémique et doit conduire à la mise en route de la chimiothérapie antituberculeuse. La prise en charge neurochirurgicale visant à une exérèse tumorale suivie du traitement anti bacillaire reste la meilleure option si la lésion est superficielle. La biopsie stéréotaxique autre technique neurochirurgicale à viser diagnostic et thérapeutique trouve son indication dans les formes profondes et les formes multiples.

## Conflits d’intérêts

Les auteurs ne déclarent aucun conflit d'intérêts.
